# 5-Bromo-2-(4-chloro­phen­yl)-3-ethyl­sulfinyl-7-methyl-1-benzofuran

**DOI:** 10.1107/S1600536810042790

**Published:** 2010-10-30

**Authors:** Hong Dae Choi, Pil Ja Seo, Byeng Wha Son, Uk Lee

**Affiliations:** a Department of Chemistry, Dongeui University, San 24 Kaya-dong Busanjin-gu, Busan 614-714, Republic of Korea; b Department of Chemistry, Pukyong National University, 599-1 Daeyeon 3-dong, Nam-gu, Busan 608-737, Republic of Korea

## Abstract

In the title compound, C_17_H_14_BrClO_2_S, the 4-chloro­phenyl ring makes a dihedral angle of 13.42 (4)° with the mean plane of the benzofuran ring. In the crystal, pairs of inter­molecular Br⋯O contacts [3.125 (1) Å] link the mol­ecules into centrosymmetric dimers, which are further linked *via* inter­molecular C—H⋯O hydrogen bonds.

## Related literature

For the pharmacological activity of benzofuran compounds, see: Aslam *et al.* (2006 ▶); Galal *et al.* (2009 ▶); Khan *et al.* (2005 ▶). For natural products with benzofuran rings, see: Akgul & Anil (2003 ▶); Soekamto *et al.* (2003 ▶). For our previous structural studies of related 3-ethyl­sulfinyl-5-halo-2-(4-halophen­yl)-7-methyl-1-benzofuran derivatives, see: Choi *et al.* (2010**a* ▶,*b* ▶,*c* ▶*,*d* ▶). For a review of halogen bonding, see: Politzer *et al.* (2007 ▶).
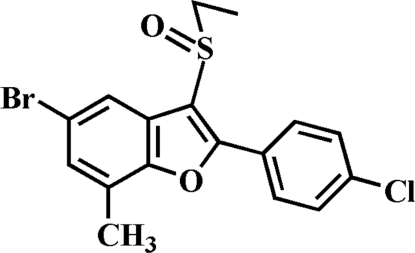


## Experimental

### 

#### Crystal data

C_17_H_14_BrClO_2_S
*M* *_r_* = 397.70
Triclinic, 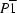

*a* = 7.3159 (1) Å
*b* = 10.3502 (2) Å
*c* = 11.8936 (2) Å
α = 68.690 (1)°
β = 89.223 (1)°
γ = 70.941 (1)°
*V* = 787.36 (2) Å^3^
*Z* = 2
Mo *K*α radiation
μ = 2.92 mm^−1^
*T* = 179 K
0.29 × 0.28 × 0.25 mm

#### Data collection

Bruker SMART APEXII CCD diffractometer
Absorption correction: multi-scan (*SADABS*; Bruker, 2009 ▶) *T*_min_ = 0.485, *T*_max_ = 0.524
14107 measured reflections
3657 independent reflections
3342 reflections with *I* > 2σ(*I*)
*R*_int_ = 0.028

#### Refinement

*R*[*F* ^2^ > 2σ(*F* ^2^)] = 0.025
*wR*(*F* ^2^) = 0.066
*S* = 1.08
3657 reflections
201 parameters
H-atom parameters constrained
Δρ_max_ = 0.44 e Å^−3^
Δρ_min_ = −0.44 e Å^−3^

### 

Data collection: *APEX2* (Bruker, 2009 ▶); cell refinement: *SAINT* (Bruker, 2009 ▶); data reduction: *SAINT*; program(s) used to solve structure: *SHELXS97* (Sheldrick, 2008 ▶); program(s) used to refine structure: *SHELXL97* (Sheldrick, 2008 ▶); molecular graphics: *ORTEP-3* (Farrugia, 1997 ▶) and *DIAMOND* (Brandenburg, 1998 ▶); software used to prepare material for publication: *SHELXL97*.

## Supplementary Material

Crystal structure: contains datablocks global, I. DOI: 10.1107/S1600536810042790/rz2506sup1.cif

Structure factors: contains datablocks I. DOI: 10.1107/S1600536810042790/rz2506Isup2.hkl

Additional supplementary materials:  crystallographic information; 3D view; checkCIF report

## Figures and Tables

**Table 1:** Hydrogen-bond geometry (Å, °)

*D*—H⋯*A*	*D*—H	H⋯*A*	*D*⋯*A*	*D*—H⋯*A*
C17—H17*B*⋯O2^i^	0.98	2.62	3.488 (2)	148

Symmetry code: (i) 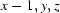.

**Fig. 1.**
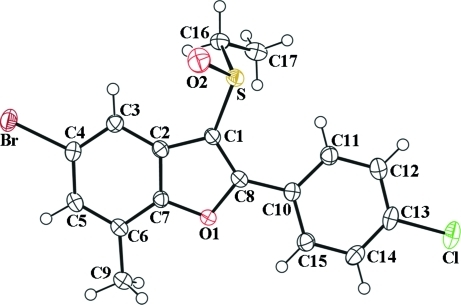
The molecular structure of the title compound with the atom numbering scheme. Displacement ellipsoids are drawn at the 50% probability level. H atoms are presented as a small spheres of arbitrary radius.

**Fig. 2.**
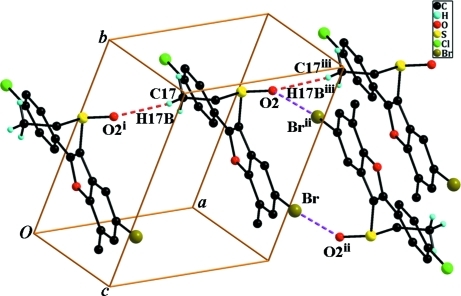
A view of the Br···O and C—H···O interactions (dashed lines) in the crystal structure of the title compound. [Symmetry codes: (i) x - 1, y, z; (ii) - x +2, - y + 1, - z + 2; (iii) x + 1, y, z.]

## supplementary crystallographic information

### Comment

Many compounds involving a benzofuran ring have received particular attention in view of their potent pharmacological properties such as antifungal, antitumor and antiviral, antimicrobial activities (Aslam *et al.*, 2006, Galal *et al.*, 2009, Khan *et al.*, 2005). These compounds widely occur in nature (Akgul & Anil, 2003; Soekamto *et al.*, 2003). As a part of our continuing studies of the substituent effect on the solid state structures of 3-ethylsulfinyl-5-halo-2-(4-halophenyl)-7-methyl-1-benzofuran analogues (Choi *et al.*, 2010*a*,*b*,*c*,d), we report herein on the crystal structure of the title compound.

In the title molecule (Fig. 1), the benzofuran unit is essentially planar, with a mean deviation of 0.019 (1) Å from the least-squares plane defined by the nine constituent atoms. The dihedral angle formed by the mean plane of the benzofuran ring and the 4-chlorophenyl ring is 13.42 (4)° . The molecular packing (Fig. 2) is stabilized by a Br···O halogen-bonding between the bromine and the oxygen of the S═O unit [Br···O2^ii^ = 3.125 (1) Å, C4—Br···O2^ii^ = 167.44 (6)° .] (Politzer *et al.*, 2007). The crystal packing (Fig. 2) is further stabilized by a weak intermolecular C—H···O hydrogen bond between the methyl H atom of the ethyl group and the S═O unit (C17—H17B···O2^i^; Table 1).

### Experimental

77% 3-chloroperoxybenzoic acid (179 mg, 0.8 mmol) was added in small portions to a stirred solution of 5-bromo-2-(4-chlorophenyl)-3-ethylsulfanyl-7-methyl-1-benzofuran (318 mg, 0.8 mmol) in dichloromethane (40 mL) at 273 K.  After being stirred at room temperature for 3h, the mixture was washed with saturated sodium bicarbonate solution and the organic layer was separated, dried over magnesium sulfate, filtered and concentrated at reduced pressure. The residue was purified by column chromatography (hexane-ethyl acetate, 2:1 v/v) to afford the title compound as a colorless solid [yield 79%, m.p. 443–444 K, *R*_f_ = 0.63 (hexane–ethyl acetate, 2:1 v/v)]. Single crystals suitable for X-ray diffraction were prepared by slow evaporation of a solution of the title compound in tetrahydrofuran at room temperature.

### Refinement

All H atoms were positioned geometrically and refined using a riding model, with C—H = 0.93 Å for aryl, 0.97 Å for methylene, and 0.96 Å for methyl H atoms. *U*_iso_(H) = 1.2*U*_eq_(C) for aryl and methylene H atoms, and 1.5*U*_eq_(C) for methyl H atoms.

### Crystal data

**Table:**

C_17_H_14_BrClO_2_S	*Z* = 2
*M**_r_* = 397.70	*F*(000) = 400
Triclinic, *P*1	*D*_x_ = 1.677 Mg m^−^^3^
Hall symbol: -P 1	Mo *K*α radiation, λ = 0.71073 Å
*a* = 7.3159 (1) Å	Cell parameters from 8401 reflections
*b* = 10.3502 (2) Å	θ = 2.3–27.6°
*c* = 11.8936 (2) Å	µ = 2.92 mm^−^^1^
α = 68.690 (1)°	*T* = 179 K
β = 89.223 (1)°	Block, colourless
γ = 70.941 (1)°	0.29 × 0.28 × 0.25 mm
*V* = 787.36 (2) Å^3^	

### Data collection

**Table:**

Bruker SMART APEXII CCD diffractometer	3657 independent reflections
Radiation source: rotating anode	3342 reflections with *I* > 2σ(*I*)
graphite multilayer	*R*_int_ = 0.028
Detector resolution: 10.0 pixels mm^-1^	θ_max_ = 27.6°, θ_min_ = 1.9°
φ and ω scans	*h* = −9→9
Absorption correction: multi-scan (*SADABS*; Bruker, 2009)	*k* = −13→13
*T*_min_ = 0.485, *T*_max_ = 0.524	*l* = −15→15
14107 measured reflections	

### Refinement

**Table:**

Refinement on *F*^2^	Primary atom site location: structure-invariant direct methods
Least-squares matrix: full	Secondary atom site location: difference Fourier map
*R*[*F*^2^ > 2σ(*F*^2^)] = 0.025	Hydrogen site location: difference Fourier map
*wR*(*F*^2^) = 0.066	H-atom parameters constrained
*S* = 1.08	*w* = 1/[σ^2^(*F*_o_^2^) + (0.0362*P*)^2^ + 0.2277*P*] where *P* = (*F*_o_^2^ + 2*F*_c_^2^)/3
3657 reflections	(Δ/σ)_max_ = 0.001
201 parameters	Δρ_max_ = 0.44 e Å^−^^3^
0 restraints	Δρ_min_ = −0.44 e Å^−^^3^

### Special details

**Table:**

Geometry. All esds (except the esd in the dihedral angle between two l.s. planes) are estimated using the full covariance matrix. The cell esds are taken into account individually in the estimation of esds in distances, angles and torsion angles; correlations between esds in cell parameters are only used when they are defined by crystal symmetry. An approximate (isotropic) treatment of cell esds is used for estimating esds involving l.s. planes.
Refinement. Refinement of F^2^ against ALL reflections. The weighted R-factor wR and goodness of fit S are based on F^2^, conventional R-factors R are based on F, with F set to zero for negative F^2^. The threshold expression of F^2^ > 2sigma(F^2^) is used only for calculating R-factors(gt) etc. and is not relevant to the choice of reflections for refinement. R-factors based on F^2^ are statistically about twice as large as those based on F, and R- factors based on ALL data will be even larger.

### Fractional atomic coordinates and isotropic or equivalent isotropic displacement parameters (Å^2^)

**Table:**

	*x*	*y*	*z*	*U*_iso_*/*U*_eq_	
Br	0.99643 (3)	0.279456 (19)	1.076951 (15)	0.03226 (7)	
Cl	0.44218 (8)	0.83326 (6)	−0.02326 (4)	0.04120 (13)	
S	0.64454 (6)	0.80596 (4)	0.58771 (4)	0.02307 (10)	
O1	0.80545 (16)	0.41969 (12)	0.55116 (10)	0.0217 (2)	
O2	0.8019 (2)	0.81168 (15)	0.66239 (13)	0.0357 (3)	
C1	0.7086 (2)	0.62059 (16)	0.60048 (14)	0.0203 (3)	
C2	0.7957 (2)	0.49198 (17)	0.71094 (15)	0.0207 (3)	
C3	0.8333 (2)	0.46726 (18)	0.83324 (15)	0.0232 (3)	
H3	0.7941	0.5455	0.8620	0.028*	
C4	0.9307 (2)	0.32235 (19)	0.91027 (15)	0.0243 (3)	
C5	0.9924 (2)	0.20505 (18)	0.87039 (16)	0.0249 (3)	
H5	1.0605	0.1081	0.9273	0.030*	
C6	0.9561 (2)	0.22752 (17)	0.74956 (15)	0.0225 (3)	
C7	0.8541 (2)	0.37343 (17)	0.67424 (14)	0.0202 (3)	
C8	0.7168 (2)	0.57118 (16)	0.50771 (15)	0.0206 (3)	
C9	1.0223 (3)	0.10666 (18)	0.70187 (17)	0.0289 (4)	
H9A	1.1085	0.0162	0.7657	0.043*	
H9B	1.0930	0.1359	0.6318	0.043*	
H9C	0.9089	0.0886	0.6769	0.043*	
C10	0.6517 (2)	0.63921 (17)	0.37745 (15)	0.0211 (3)	
C11	0.5182 (2)	0.78327 (18)	0.32282 (16)	0.0253 (3)	
H11	0.4701	0.8401	0.3710	0.030*	
C12	0.4555 (2)	0.84388 (19)	0.19966 (16)	0.0280 (4)	
H12	0.3666	0.9422	0.1628	0.034*	
C13	0.5241 (3)	0.7592 (2)	0.13105 (15)	0.0277 (4)	
C14	0.6558 (3)	0.6165 (2)	0.18176 (16)	0.0282 (4)	
H14	0.7013	0.5602	0.1329	0.034*	
C15	0.7202 (2)	0.55713 (18)	0.30474 (15)	0.0251 (3)	
H15	0.8118	0.4596	0.3403	0.030*	
C16	0.4362 (3)	0.81428 (19)	0.67208 (17)	0.0283 (4)	
H16A	0.4677	0.7232	0.7463	0.034*	
H16B	0.4054	0.8995	0.6974	0.034*	
C17	0.2605 (3)	0.8300 (2)	0.59514 (19)	0.0319 (4)	
H17A	0.2222	0.9245	0.5255	0.048*	
H17B	0.1522	0.8268	0.6442	0.048*	
H17C	0.2936	0.7489	0.5660	0.048*	

### Atomic displacement parameters (Å^2^)

**Table:**

	*U*^11^	*U*^22^	*U*^33^	*U*^12^	*U*^13^	*U*^23^
Br	0.04398 (12)	0.02976 (11)	0.02033 (10)	−0.01382 (8)	−0.00429 (7)	−0.00526 (7)
Cl	0.0456 (3)	0.0526 (3)	0.0191 (2)	−0.0187 (2)	−0.00070 (18)	−0.0048 (2)
S	0.02667 (19)	0.01856 (18)	0.0238 (2)	−0.00802 (15)	0.00110 (15)	−0.00753 (15)
O1	0.0249 (5)	0.0178 (5)	0.0201 (6)	−0.0043 (4)	0.0014 (4)	−0.0073 (4)
O2	0.0393 (7)	0.0338 (7)	0.0379 (8)	−0.0181 (6)	−0.0048 (6)	−0.0127 (6)
C1	0.0203 (7)	0.0179 (7)	0.0206 (8)	−0.0052 (6)	0.0015 (6)	−0.0061 (6)
C2	0.0185 (7)	0.0198 (7)	0.0224 (8)	−0.0061 (6)	0.0017 (6)	−0.0067 (6)
C3	0.0248 (7)	0.0228 (8)	0.0222 (8)	−0.0082 (6)	0.0009 (6)	−0.0087 (6)
C4	0.0248 (7)	0.0270 (8)	0.0201 (8)	−0.0099 (6)	−0.0007 (6)	−0.0068 (6)
C5	0.0238 (7)	0.0198 (7)	0.0253 (8)	−0.0054 (6)	−0.0016 (6)	−0.0037 (6)
C6	0.0200 (7)	0.0192 (7)	0.0256 (8)	−0.0054 (6)	0.0012 (6)	−0.0068 (6)
C7	0.0195 (7)	0.0208 (7)	0.0190 (7)	−0.0061 (6)	0.0005 (6)	−0.0068 (6)
C8	0.0191 (7)	0.0173 (7)	0.0233 (8)	−0.0053 (6)	0.0022 (6)	−0.0063 (6)
C9	0.0305 (8)	0.0222 (8)	0.0299 (9)	−0.0039 (7)	0.0014 (7)	−0.0099 (7)
C10	0.0210 (7)	0.0226 (7)	0.0205 (8)	−0.0104 (6)	0.0028 (6)	−0.0064 (6)
C11	0.0242 (8)	0.0261 (8)	0.0233 (8)	−0.0074 (6)	0.0018 (6)	−0.0080 (7)
C12	0.0250 (8)	0.0277 (8)	0.0252 (9)	−0.0085 (7)	0.0008 (7)	−0.0038 (7)
C13	0.0293 (8)	0.0364 (9)	0.0167 (8)	−0.0171 (7)	0.0022 (6)	−0.0040 (7)
C14	0.0347 (9)	0.0330 (9)	0.0223 (8)	−0.0164 (7)	0.0080 (7)	−0.0124 (7)
C15	0.0286 (8)	0.0233 (8)	0.0234 (8)	−0.0102 (6)	0.0044 (6)	−0.0080 (7)
C16	0.0308 (8)	0.0249 (8)	0.0272 (9)	−0.0046 (7)	0.0060 (7)	−0.0120 (7)
C17	0.0288 (8)	0.0286 (9)	0.0413 (11)	−0.0105 (7)	0.0088 (8)	−0.0163 (8)

### Geometric parameters (Å, °)

**Table:**

Br—C4	1.8987 (17)	C9—H9A	0.9800
Br—O2^i^	3.1254 (14)	C9—H9B	0.9800
Cl—C13	1.7385 (17)	C9—H9C	0.9800
S—O2	1.4925 (13)	C10—C11	1.400 (2)
S—C1	1.7683 (16)	C10—C15	1.402 (2)
S—C16	1.8092 (18)	C11—C12	1.383 (2)
O1—C7	1.3756 (19)	C11—H11	0.9500
O1—C8	1.3792 (18)	C12—C13	1.381 (3)
C1—C8	1.368 (2)	C12—H12	0.9500
C1—C2	1.450 (2)	C13—C14	1.383 (3)
C2—C7	1.386 (2)	C14—C15	1.384 (2)
C2—C3	1.397 (2)	C14—H14	0.9500
C3—C4	1.385 (2)	C15—H15	0.9500
C3—H3	0.9500	C16—C17	1.518 (3)
C4—C5	1.399 (2)	C16—H16A	0.9900
C5—C6	1.385 (2)	C16—H16B	0.9900
C5—H5	0.9500	C17—H17A	0.9800
C6—C7	1.392 (2)	C17—H17B	0.9800
C6—C9	1.495 (2)	C17—H17C	0.9800
C8—C10	1.460 (2)		
			
C4—Br—O2^i^	167.44 (6)	H9A—C9—H9C	109.5
O2—S—C1	106.73 (7)	H9B—C9—H9C	109.5
O2—S—C16	107.42 (9)	C11—C10—C15	118.61 (15)
C1—S—C16	98.10 (8)	C11—C10—C8	122.03 (15)
C7—O1—C8	106.72 (12)	C15—C10—C8	119.34 (14)
C8—C1—C2	107.07 (14)	C12—C11—C10	120.86 (16)
C8—C1—S	127.14 (12)	C12—C11—H11	119.6
C2—C1—S	124.97 (12)	C10—C11—H11	119.6
C7—C2—C3	119.41 (14)	C13—C12—C11	118.98 (16)
C7—C2—C1	105.01 (14)	C13—C12—H12	120.5
C3—C2—C1	135.55 (15)	C11—C12—H12	120.5
C4—C3—C2	116.31 (15)	C12—C13—C14	121.83 (16)
C4—C3—H3	121.8	C12—C13—Cl	119.27 (14)
C2—C3—H3	121.8	C14—C13—Cl	118.90 (15)
C3—C4—C5	123.16 (16)	C13—C14—C15	118.96 (17)
C3—C4—Br	119.20 (13)	C13—C14—H14	120.5
C5—C4—Br	117.57 (12)	C15—C14—H14	120.5
C6—C5—C4	121.28 (15)	C14—C15—C10	120.75 (16)
C6—C5—H5	119.4	C14—C15—H15	119.6
C4—C5—H5	119.4	C10—C15—H15	119.6
C5—C6—C7	114.60 (15)	C17—C16—S	110.69 (13)
C5—C6—C9	123.38 (14)	C17—C16—H16A	109.5
C7—C6—C9	122.01 (15)	S—C16—H16A	109.5
O1—C7—C2	110.87 (13)	C17—C16—H16B	109.5
O1—C7—C6	123.88 (14)	S—C16—H16B	109.5
C2—C7—C6	125.19 (15)	H16A—C16—H16B	108.1
C1—C8—O1	110.31 (13)	C16—C17—H17A	109.5
C1—C8—C10	135.56 (14)	C16—C17—H17B	109.5
O1—C8—C10	114.12 (13)	H17A—C17—H17B	109.5
C6—C9—H9A	109.5	C16—C17—H17C	109.5
C6—C9—H9B	109.5	H17A—C17—H17C	109.5
H9A—C9—H9B	109.5	H17B—C17—H17C	109.5
C6—C9—H9C	109.5		
			
O2—S—C1—C8	−129.10 (15)	C9—C6—C7—O1	0.0 (2)
C16—S—C1—C8	119.89 (15)	C5—C6—C7—C2	2.2 (2)
O2—S—C1—C2	39.12 (15)	C9—C6—C7—C2	−177.01 (15)
C16—S—C1—C2	−71.89 (15)	C2—C1—C8—O1	−0.72 (17)
C8—C1—C2—C7	1.23 (17)	S—C1—C8—O1	169.20 (11)
S—C1—C2—C7	−168.97 (12)	C2—C1—C8—C10	177.77 (16)
C8—C1—C2—C3	179.21 (17)	S—C1—C8—C10	−12.3 (3)
S—C1—C2—C3	9.0 (3)	C7—O1—C8—C1	−0.09 (16)
C7—C2—C3—C4	0.8 (2)	C7—O1—C8—C10	−178.93 (12)
C1—C2—C3—C4	−176.99 (17)	C1—C8—C10—C11	−14.7 (3)
C2—C3—C4—C5	0.8 (2)	O1—C8—C10—C11	163.78 (14)
C2—C3—C4—Br	177.69 (11)	C1—C8—C10—C15	167.14 (18)
O2^i^—Br—C4—C3	−115.1 (3)	O1—C8—C10—C15	−14.4 (2)
O2^i^—Br—C4—C5	62.0 (3)	C15—C10—C11—C12	−0.3 (2)
C3—C4—C5—C6	−1.0 (3)	C8—C10—C11—C12	−178.48 (15)
Br—C4—C5—C6	−177.90 (12)	C10—C11—C12—C13	1.1 (3)
C4—C5—C6—C7	−0.5 (2)	C11—C12—C13—C14	−1.0 (3)
C4—C5—C6—C9	178.70 (16)	C11—C12—C13—Cl	178.70 (13)
C8—O1—C7—C2	0.92 (16)	C12—C13—C14—C15	0.1 (3)
C8—O1—C7—C6	−176.43 (15)	Cl—C13—C14—C15	−179.60 (13)
C3—C2—C7—O1	−179.71 (13)	C13—C14—C15—C10	0.7 (3)
C1—C2—C7—O1	−1.33 (17)	C11—C10—C15—C14	−0.7 (2)
C3—C2—C7—C6	−2.4 (2)	C8—C10—C15—C14	177.61 (15)
C1—C2—C7—C6	175.98 (15)	O2—S—C16—C17	172.31 (12)
C5—C6—C7—O1	179.15 (14)	C1—S—C16—C17	−77.25 (13)

Symmetry codes: (i) −*x*+2, −*y*+1, −*z*+2.

### Hydrogen-bond geometry (Å, °)

**Table:**

*D*—H···*A*	*D*—H	H···*A*	*D*···*A*	*D*—H···*A*
C17—H17B···O2^ii^	0.98	2.62	3.488 (2)	148

Symmetry codes: (ii) *x*−1, *y*, *z*.
